# Next-generation sequencing in the clinical genetic screening of patients with pheochromocytoma and paraganglioma

**DOI:** 10.1530/EC-13-0009

**Published:** 2013-05-28

**Authors:** Joakim Crona, Alberto Delgado Verdugo, Dan Granberg, Staffan Welin, Peter Stålberg, Per Hellman, Peyman Björklund

**Affiliations:** 1 Department of Surgical Sciences Uppsala University S-751 85, Uppsala Sweden; 2 Department of Medical Sciences Uppsala University S-751 85, UppsalaSweden

**Keywords:** exome sequencing, whole genome sequencing, pheochromocytoma, paraganglioma

## Abstract

**Background:**

Recent findings have shown that up to 60% of pheochromocytomas (PCCs) and paragangliomas (PGLs) are caused by germline or somatic mutations in one of the 11 hitherto known susceptibility genes: *SDHA*, *SDHB*, *SDHC*, *SDHD*, *SDHAF2*, *VHL*, *HIF2A* (*EPAS1*), *RET*, *NF1*, *TMEM127* and *MAX*. This list of genes is constantly growing and the 11 genes together consist of 144 exons. A genetic screening test is extensively time consuming and expensive. Hence, we introduce next-generation sequencing (NGS) as a time-efficient and cost-effective alternative.

**Methods:**

Tumour lesions from three patients with apparently sporadic PCC were subjected to whole exome sequencing utilizing Agilent Sureselect target enrichment system and Illumina Hi seq platform. Bioinformatics analysis was performed in-house using commercially available software. Variants in PCC and PGL susceptibility genes were identified.

**Results:**

We have identified 16 unique genetic variants in PCC susceptibility loci in three different PCC, spending less than a 30-min hands-on, in-house time. Two patients had one unique variant each that was classified as probably and possibly pathogenic: NF1 Arg304Ter and RET Tyr791Phe. The *RET* variant was verified by Sanger sequencing.

**Conclusions:**

NGS can serve as a fast and cost-effective method in the clinical genetic screening of PCC. The bioinformatics analysis may be performed without expert skills. We identified process optimization, characterization of unknown variants and determination of additive effects of multiple variants as key issues to be addressed by future studies.

## Introduction

Pheochromocytomas (PCCs) and paragangliomas (PGLs) are rare tumours arising from chromaffin cells in adrenal medulla and autonomous ganglia. A majority of these tumours have a low proliferation and seldom metastasize. The understanding of underlying molecular mechanisms in the tumorigenesis of these diseases has increased dramatically during the last decade (1). Up to 80% of all PCC and PGL could have either germline or somatic mutations (2, 3, 4) in one of the 11 hitherto known susceptibility genes: *SDHA*, *SDHB*, *SDHC*, *SDHD*, *SDHAF2*, *VHL*, *HIF2A* (*EPAS1*), *RET*, *NF1*, *TMEM127* and *MAX* (5, 6, 7, 8, 9, 10, 11). While there has been a constant flow of reported new susceptibility loci, the capacity of instruments approved for diagnostic use has failed to keep up with the increasing demand. These 11 genes constitute 144 exons (∼25 000 bases); consequently, a comprehensive PCC and PGL genetic screening test can be time consuming and is not regarded as cost effective (12). This has motivated the design of numerous screening algorithms to guide the investigators in the selection of appropriate patients and tests (12, 13). Spare use of clinical genetic screening in patients with PCC and PGL, despite the introduction of such guidelines, has been mainly excused by cost–benefit explanations.

Introduction of novel sequencing techniques (denoted next-generation sequencing or NGS) has dramatically reduced the cost for DNA sequencing (14). The term NGS includes principally different sequencing platforms that share a high output of sequenced bases relative to traditional methods. Recently, the focus of experiments using NGS has been shifted from the research settings to investigate the use of NGS as a platform in clinical scenarios (15, 16, 17, 18).

The NGS process is highly complex with multiple steps that may be divided into genomic enrichment (selected, all exons as in exome or none as in whole genome sequencing), sequencing (including library preparation), bioinformatics analysis and, in the clinical setting, genetic consultation (19).

Due to its well-characterized genotype–phenotype correlation and the limitations imposed by existing technologies, there is a strong argument for investigating the potential use of NGS as a diagnostic test in the clinical genetic screening of PCC and PGL.

## Materials and methods

### Patients

Tumour tissues from three patients with PCC were selected for whole exome sequencing. Patient characteristics are summarized in Table 1. All the three patients had a secretory unilateral PCC and no apparent signs/symptoms/history suggesting pathogenic germline variants in known susceptibility genes. The local ethics committee approved the study and written informed consent was obtained from all patients.

### Exome capture and high-throughput sequencing

All samples were macro-dissected to achieve neoplastic cellularity of >80%. DNA was prepared from cryosections using Genomic-tip 20/G (cat. no. 10223, Qiagen). Sequencing libraries were prepared from 3 μg gDNA using SureSelect target enrichment system for Illumina paired-end sequencing libraries v2.2, October 2010 (Agilent Technologies, Santa Clara, CA, USA), according to the manufacturer's instructions. Briefly, the DNA was fragmented using the Covaris S2 system (Covaris, Woburn, MA, USA). The DNA fragments were end-repaired using T4 DNA polymerase, Klenow DNA polymerase and T4 polynucleotide kinase (PNK), followed by purification using AMPure XP beads (Beckman Coulter, Brea, CA, USA). An A-base was ligated to the blunt ends of the DNA fragments using the Klenow DNA polymerase and the sample was purified using AMPure XP beads. Adapters for sequencing were ligated to the DNA fragments, followed by purification using AMPure XP beads. The adapter-ligated libraries were amplified for five PCR cycles, followed by a second purification using AMPure XP beads. The quality of the enriched libraries was evaluated using the 2100 Bioanalyzer and a DNA 1000 kit (Agilent). Exon capture was performed from 500 ng of each sequencing library using the SureSelect Human All Exon 50 Mb kit (Agilent). Briefly, the fragments in the library were hybridized to capture probes, unhybridized material was washed away and the captured fragments were amplified for ten PCR cycles, followed by purification using AMPure XP beads. The quality of the enriched libraries was evaluated using the 2100 Bioanalyzer and a High-Sensitivity DNA-kit (Agilent). The adapter-ligated fragments were quantified by qPCR using the KAPA SYBR FAST library quantification kit for Illumina Genome Analyzer (KAPA Biosystems, Woburn, MA, USA). A 6 pM solution of the sequencing libraries was subjected to cluster generation on the cBot instrument (Illumina, Inc., San Diego, CA, USA). Paired-end sequencing was performed for 100 cycles in one lane using a HiSeq2000 instrument (Illumina, Inc.), according to the manufacturer's protocols. Base calling was done on the same instrument by RTA 1.10.36 and the resulting bcl files were converted to Illumina qseq format with tools provided by OLB-1.9.0 (Illumina, Inc.). Fastq sequence files were generated using CASAVA 1.7.0 (Illumina, Inc.). Additional statistics on sequence quality was compiled from the base call files with an in-house script (http://molmed.medsci.uu.se/SNP+SEQ+Technology+Platform/).

### Bioinformatics

**Figure 1:**
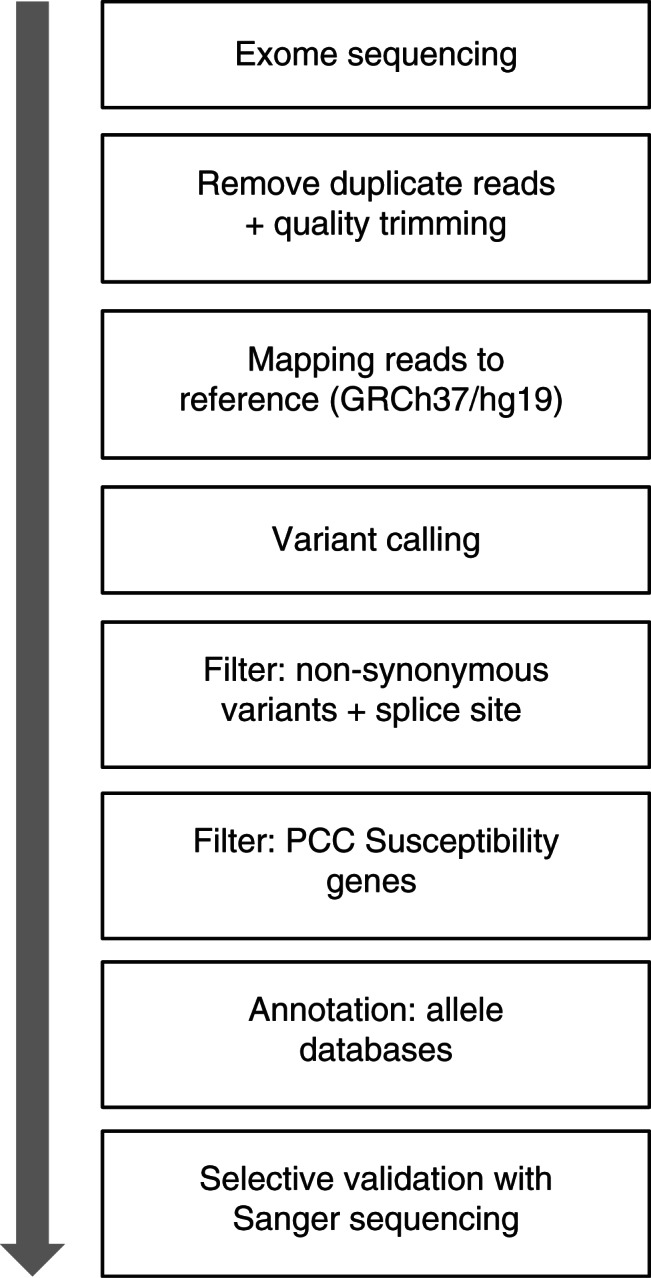
Bioinformatics pipeline for analysis of exome sequencing in the clinical genetic screening of pheochromocytoma.

Sequencing generated a minimum of 125×10^6^ reads in all three tumours with an average read length of 100 reads (Table 2). Generated sequences were processed using commercially available software: CLC Genomics Workbench 4.9 (CLC Bio, Aarhus, Denmark). Reads from pair-end fragments were trimmed for low-quality and duplicate reads (Fig. 1). Remaining sequences were mapped to the human reference sequence GRCh37.p5. A single-nucleotide variant (SNV) and insertion/deletion detection algorithm was used with low- and high-stringency settings: low stringency, coverage of >8 reads and a variant allele frequency of >25%; and high stringency, coverage of >30 reads and a variant allele frequency of >35%. Generated results were filtered for non-synonymous variants and/or variants with a probable splice site effect. The list was annotated for all gene annotations and then filtered for variants in one of the 11 currently known PCC susceptibility genes. The remaining variants were annotated for overlapping information in selected genetic databases: the Single Nucleotide Polymorphism Database (dbSNP), Catalogue of Somatic Mutations in Cancer (COSMIC), the Human Gene Mutation Database (HGMD) and Leiden Open source Variation Databases (LOVD). Impact of non-synonymous amino acid substitution was assessed *in silico*, using Polyphen2 (20) and SIFT (21). Cross-references were manually gathered when available. Analysis of structural variants in data generated by exome sequencing was not adequately supported by the software and was excluded from this experiment.

### Sanger sequencing

DNA was prepared from peripheral blood and tumour cryosections using DNeasy Blood and Tissue Kit (Qiagen). In order to be utilized as control and for verification of variants discovered by NGS, fragments corresponding to all exons and intron–exon junctions of major susceptibility genes; *SDHB*, *SDHC*, *VHL*, *MAX*, *RET* (exons 10, 11 and 13–16) as well as selected fragments in *NF1* (exon 9), were amplified by PCR and sequenced using automated Sanger sequencing (Beckman Coulter, Takeley, UK). Primer sequences and PCR conditions can be obtained by request.

## Results

**Figure 2:**
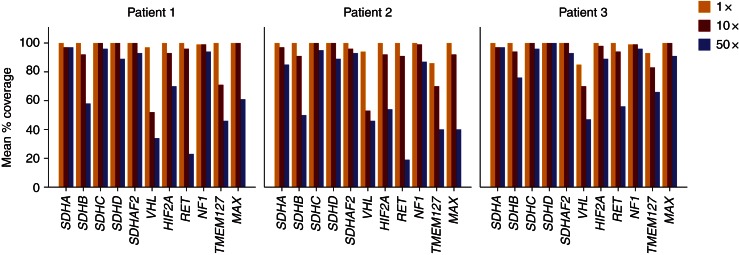
Detailed coverage at bases annotated for PCC susceptibility genes.

Exome sequencing of three PCC tumour lesions generated a read coverage of 1× (98–99%), 10× (94–96%) and 100× (35–77%) for bases annotated by PCC susceptibility genes (Table 2 and Fig. 2). A total of 30 and 19 variants were identified with low and high variant-calling stringency respectively (Supplementary Table 1, see section on supplementary data given at the end of this article). In low stringency, this corresponded to 16 unique variants. One was assessed as probably pathogenic, one as possibly pathogenic, four as benign and 11 as unknown. RET Tyr791Phe and NF1 Arg304Ter were each found in one patient and were assessed as either possibly or probably pathogenic. Patient 1 had all variants classified as benign or unknown, including one previously uncharacterized variant in *SDHC*: Pro110Ser (Supplementary Figure 1, 2 and 3, see section on supplementary data given at the end of this article). RET Tyr791Phe and SDHC Pro110Ser were verified by Sanger sequencing in both blood and tumour tissues. Comparing with results from Sanger sequencing of *SDHB*, *SDHC*, *VHL*, *RET* (exons 10–11 and 13–16) and *MAX* as control, there were no false negatives generated by NGS (Supplementary Table 1).

### Patient 1: *SDHC* variant of uncertain clinical significance

A 61-year-old woman was investigated due to therapy-resistant hypertension of unknown aetiology. Urine noradrenaline level was elevated. The patient was operated with a laparoscopic left-sided adrenalectomy and the pathology report described a benign PCC, 25×20 mm in size and a weight of 4.5 g. Immunohistochemistry demonstrated expression for chromogranin A and a Ki67 index of 1%. Exome sequencing revealed seven SNVs, one was classified as benign and six as unknown. There was one missense variant in *SDHC* located at position 477C<T, resulting in amino acid substitution Pro110Ser. This variant was not found in the HDMD, dbSNP, COSMIC or LOVD databases nor could it be found in a PubMed search. *In silico* analysis using Polyphen2 and SIFT estimated SDHC Pro110Ser as benign (score 0.231) and tolerated (score 0.93).

### Patient 2: *RET* variant of uncertain clinical significance

**Figure 3:**
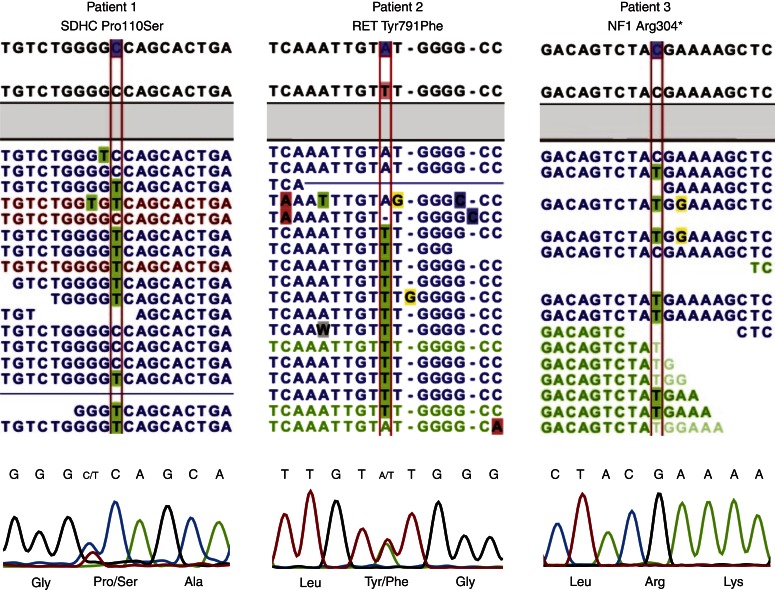
Screenshot of sequences as displayed in CLC genomics 4.9. From above: reference sequence, consensus sequence and mapped tumour reads (blue colour, intact read pairs; green colour, broken forward read; red colour, broken reverse read). Below: chromatograms of the corresponding sequences generated by Sanger sequencing.

A 27-year-old woman was investigated *post partum* due to therapy-resistant hypertension during the second and third trimesters. The patient had elevated urine noradrenaline and adrenaline levels. She was operated with a laparoscopic right-sided adrenalectomy and the pathology report described a PCC, 50×50 mm in size with a weight of 54 g. Immunohistochemistry showed strong staining of chromogranin A and a Ki67 index of <0.5%. Exome sequencing revealed 13 SNVs, three were classified as benign and nine as unknown. One missense variant was assessed as possibly pathogenic, located at position 2372A<T (rs77724903), resulting in the amino acid substitution Tyr791Phe, in the proto-oncogene tyrosine-protein kinase receptor (*RET*) gene (Fig. 3). The pathogenicity of RET Tyr791Phe is disputed (22, 23, 24).

### Patient 3: *NF1* variant

A 65-year-old woman with a two-decade history of hypertension and newly diagnosed adenocarcinoma of the breast was investigated due to abdominal discomfort. Computed tomography of the abdomen showed a lesion in the left adrenal gland and subsequent urine collection revealed high levels of noradrenaline. The patient was operated with a left-sided adrenalectomy and the pathology report described a cystic PCC, 60×50 mm in size and a weight of 59 g. The immunoreactivity of chromogranin A was strong and Ki67 index was <1%. Exome sequencing revealed ten SNVs, nine were classified as unknown. One missense variant was assessed as probably pathogenic, a nonsense variant located at position 910C>T (rs76015786), resulting in the amino acid substitution Arg304Ter, in the neurofibromin (*NF1*) gene. The phenotype of Arg304Ter is described in related tumours and we assessed the variants as probably pathogenic (25, 26, 27). However, this variation could not be confirmed by Sanger sequencing.

## Discussion

Genetic screening of PCC and PGL has been found to be beneficial in practicing centres (28). Utilizing novel sequencing techniques have a potential to decrease costs and time consumption, thereby lowering the threshold for inclusion.

Finding of the clinically relevant allele RET Tyr791Phe clearly exemplified the potential of NGS as a diagnostic tool, while SDHC Pro110Ser illustrated the complexity of possibly pathogenic, but previously unknown, variants. NF1 Arg304Ter displayed potential methodology conflicts; however, conflicts in results generated by certified clinical genetic laboratory testing using Sanger sequencing have been reported (29).

### Price

A direct cost comparison between whole exome sequencing and traditional methods is complicated due to the invariability in which genetic screening is currently performed. The total cost for analysing the most frequently mutated genes (*SDHB*, *SDHD*, *VHL* and *RET*) is estimated to be 3500 USD (12, 30) and if screening all ten susceptibility genes, we estimate the cost to be 10 000 USD. The use of genetic screening algorithms may clearly reduce costs but can be time consuming and are designed for scenarios in which patient characteristics clearly indicate specific loci (31). The costs of exome enrichment and sequencing in this study were considerably lower than those of traditional screening, and as the techniques develop fast, further cost reductions are expected (Hayden EC, The $1000 genome: are we there yet?, 2012, NATURE NEWS BLOG).

### Performance

Raw sequences generated by NGS require computational processing, mapping reads to a reference sequence and calling variants between the two. Results generated by NGS should be confirmed with a principally different sequencing chemistry. The bioinformatics process should deliver a defined list of variants. Stochastic false positives occur at relatively high frequencies but may be filtered given that the position is covered by an adequate sequence depth (about 30-fold). False negatives are more insidious and may be caused by incomplete enrichment, uneven sequencing coverage or faulty bioinformatics processing (17). Additionally, a high sequence depth allows NGS to detect alleles at thresholds below that of Sanger sequencing. These specifications predict built-in conflicts in which NGS may generate probably pathological variants that cannot be validated by Sanger sequencing (i.e. patient 3). Other validation methods (e.g. pyrosequencing) may detect alleles at a lower frequency but at a higher cost (32). A situation with multiple unknown variants has been expected and was confirmed by this study (1). Evaluating the significance of such ‘genetic incidentalomas’ may be extensively recourse demanding and clearly demonstrates the need to further expand and curate allele databases such as dbSNP and LOVD.

Time constraint in a clinical setting is also a challenge. A diagnostic test must have a throughput measurable in weeks. In theory, the NGS process can be tuned to deliver results within 1 week (33). With a pre-defined bioinformatics assay, the necessary computational analysis for our experiments had a throughput of <24 h, including a total in-house hands-on time of <30 min.

### Exome vs targeted enrichment

Sequencing of tumour tissue with complete exome coverage differs from the current diagnostic procedure in which limited loci in germline DNA is analysed. The theoretical potential is to provide improved prognostic and/or predictive information to individualize the care of the patient (34, 35). Managing the surplus of genetic information that does not involve genes associated with the specific disease nor with its treatment is problematic (36). Ethical and financial frameworks regarding rights and responsibilities of patients and providers need to be implemented (16). While the concept of personalized medicine based on whole genome or exome coverage needs to mature, there are immediate benefits of NGS in clinical situations such as in the PCC and PGL patients. Examining the available sequencing apparatus and the upcoming pipeline, applications classified as medium capacity are closest to fulfilling the optimal specification of requirements for this situation: low costs, fast throughput, high accuracy and a capacity matching the size of loci conferring susceptibility to PCC and PGL (35, 37).

### Limitations of this study

Exome enrichment resulted in a coverage of above ten reads for more than 90% of targeted regions. However, detailed coverage analysis (Fig. 2) revealed PCC loci lacking 10× coverage (*VHL* gene had 10× coverage at only ∼50% of bases). Use of exome enrichment prevents analysis of structural variants (38), thus limiting the comparison of NGS results with current standards (Multiplex Ligation-dependent Probe Amplification).

As tumour tissue was sequenced without matched constitutional DNA, the bioinformatics process could not classify variants as somatic or constitutional. Therefore, future studies should include multiple cases with matched tumoral and normal tissues from patients having characterized pathogenic disease-causing variants. The method for target enrichment should be selected with regard to expected coverage at PCC and PGL disease causing loci.

Sanger sequencing as a validation method for NGS results have been replaced by other more sensitive methods (39); the finding of NF1 Arg304Ter by NGS, but not by Sanger, is an example of inconclusiveness between these two methods.

## Conclusion

We conclude that utilizing NGS may serve as a fast and cost-effective method in the clinical genetic screening of patients with PCC and PGLs. In order to facilitate the introduction of NGS as a diagnostic application, we identified process optimization, characterization of unknown variants and determination of additive effects of multiple variants as key issues to be addressed by future studies.

## Supplementary data

This is linked to the online version of the paper at http://dx.doi.org/10.1530/EC-13-0009.

## Figures and Tables

**Table 1:** Clinical characteristics of sequenced patients

**Patient no.**	**Sex**	**Age at diagnosis**	**Pre-operative characteristics**				**Post-operative characteristics**				
Symptoms of PCC/PGL syndrome	Family history	Size (mm)	Bilateral/multiple lesions	Diagnosis	Ki67 (%)	Survival (follow up)	Metastatic disease	Recurrent disease
1	F	61	No	No	25	Unilateral	PCC	<1	(75)	No	No
2	F	27	No	No	100	Unilateral	PCC	<1	(56)	No	No
3	F	66	No	No	60	Unilateral	PCC	<1	(66)	No	No

Survival in months. F, female; M, male; family history, one first-degree relative or two second-degree relatives; PCC, pheochromocytomas.

**Table 2:** Quality scores of whole exome sequencing

**Tumour id#**	**No. of reads**		**Average read length**	**Whole exome, coding sequences**			**Coding sequence PCC susceptibility genes**				**Total no. of variants; non-synonymous and splice site**	
Pre-trim	Post-trim		10× coverage (%)	Non-synonymous variants	Small INDELs	Mean coverage	1× (%)	10× (%)	100× (%)	Low stringency	High stringency
1	126×10^6^	86×10^6^	100	92	15 240	230	93	98	94	35	13	8
2	129×10^6^	112×10^6^	100	95	14 527	222	127	99	95	57	8	5
3	243×10^6^	206×10^6^	100	94	17 051	252	198	99	96	77	10	5

No., number; Trim, removal of duplicate and low-quality reads; PCC susceptibility genes, *SDHA*, *SDHB*, *SDHC*, *SDHD*, *SDHAF2*, *VHL*, *HIF2A*, *RET*, *NF1*, *TMEM127* and *MAX*; PCC, pheochromocytoma.

## References

[1] Gimenez-Roqueplo AP, Dahia PL, Robledo M (2012). An update on the genetics of paraganglioma, pheochromocytoma, and associated hereditary syndromes. Hormone and Metabolic Research.

[2] Cascon A, Pita G, Burnichon N, Landa I, Lopez-Jimenez E, Montero-Conde C, Leskela S, Leandro-Garcia LJ, Leton R, Rodriguez-Antona C (2009). Genetics of pheochromocytoma and paraganglioma in Spanish patients. Journal of Clinical Endocrinology and Metabolism.

[3] Burnichon N, Vescovo L, Amar L, Libe R, de Reynies A, Venisse A, Jouanno E, Laurendeau I, Parfait B, Bertherat J (2011). Integrative genomic analysis reveals somatic mutations in pheochromocytoma and paraganglioma. Human Molecular Genetics.

[4] Burnichon N, Buffet A, Parfait B, Letouze E, Laurendeau I, Loriot C, Pasmant E, Abermil N, Valeyrie-Allanore L, Bertherat J (2012). Somatic NF1 inactivation is a frequent event in sporadic pheochromocytoma. Human Molecular Genetics.

[5] Burnichon N, Briere JJ, Libe R, Vescovo L, Riviere J, Tissier F, Jouanno E, Jeunemaitre X, Benit P, Tzagoloff A (2010). SDHA is a tumor suppressor gene causing paraganglioma. Human Molecular Genetics.

[6] Comino-Mendez I, Gracia-Aznarez FJ, Schiavi F, Landa I, Leandro-Garcia LJ, Leton R, Honrado E, Ramos-Medina R, Caronia D, Pita G (2011). Exome sequencing identifies MAX mutations as a cause of hereditary pheochromocytoma. Nature Genetics.

[7] Gimenez-Roqueplo AP, Lehnert H, Mannelli M, Neumann H, Opocher G, Maher ER, Plouin PF (2006). Phaeochromocytoma, new genes and screening strategies. Clinical Endocrinology.

[8] Hao HX, Khalimonchuk O, Schraders M, Dephoure N, Bayley JP, Kunst H, Devilee P, Cremers CW, Schiffman JD, Bentz BG (2009). SDH5, a gene required for flavination of succinate dehydrogenase, is mutated in paraganglioma. Science.

[9] Neumann HP, Bausch B, McWhinney SR, Bender BU, Gimm O, Franke G, Schipper J, Klisch J, Altehoefer C, Zerres K (2002). Germ-line mutations in nonsyndromic pheochromocytoma. New England Journal of Medicine.

[10] Yao L, Schiavi F, Cascon A, Qin Y, Inglada-Perez L, King EE, Toledo RA, Ercolino T, Rapizzi E, Ricketts CJ (2010). Spectrum and prevalence of FP/TMEM127 gene mutations in pheochromocytomas and paragangliomas. Journal of the American Medical Association.

[11] Zhuang Z, Yang C, Lorenzo F, Merino M, Fojo T, Kebebew E, Popovic V, Stratakis CA, Prchal JT, Pacak K (2012). Somatic HIF2A gain-of-function mutations in paraganglioma with polycythemia. New England Journal of Medicine.

[12] Erlic Z, Rybicki L, Peczkowska M, Golcher H, Kann PH, Brauckhoff M, Mussig K, Muresan M, Schaffler A, Reisch N (2009). Clinical predictors and algorithm for the genetic diagnosis of pheochromocytoma patients. Clinical Cancer Research.

[13] Karasek D, Frysak Z, Pacak K (2010). Genetic testing for pheochromocytoma. Current Hypertension Reports.

[14] Bick D, Dimmock D (2011). Whole exome and whole genome sequencing. Current Opinion in Pediatrics.

[15] Norton N, Li D, Hershberger RE (2012). Next-generation sequencing to identify genetic causes of cardiomyopathies. Current Opinion in Cardiology.

[16] Ku CS, Cooper DN, Polychronakos C, Naidoo N, Wu M, Soong R (2012). Exome sequencing: dual role as a discovery and diagnostic tool. Annals of Neurology.

[17] Montenegro G, Powell E, Huang J, Speziani F, Edwards YJ, Beecham G, Hulme W, Siskind C, Vance J, Shy M (2011). Exome sequencing allows for rapid gene identification in a Charcot-Marie-Tooth family. Annals of Neurology.

[18] Artuso R, Fallerini C, Dosa L, Scionti F, Clementi M, Garosi G, Massella L, Epistolato MC, Mancini R, Mari F (2012). Advances in Alport syndrome diagnosis using next-generation sequencing. European Journal of Human Genetics.

[19] Haas J, Katus HA, Meder B (2011). Next-generation sequencing entering the clinical arena. Molecular and Cellular Probes.

[20] Adzhubei IA, Schmidt S, Peshkin L, Ramensky VE, Gerasimova A, Bork P, Kondrashov AS, Sunyaev SR (2010). A method and server for predicting damaging missense mutations. Nature Methods.

[21] Kumar P, Henikoff S, Ng PC (2009). Predicting the effects of coding non-synonymous variants on protein function using the SIFT algorithm. Nature Protocols.

[22] Frank-Raue K, Machens A, Scheuba C, Niederle B, Dralle H, Raue F (2008). Difference in development of medullary thyroid carcinoma among carriers of RET mutations in codons 790 and 791. Clinical Endocrinology.

[23] Vaclavikova E, Dvorakova S, Sykorova V, Bilek R, Dvorakova K, Vlcek P, Skaba R, Zelinka T, Bendlova B (2009). RET mutation Tyr791Phe: the genetic cause of different diseases derived from neural crest. Endocrine.

[24] Erlic Z, Hoffmann MM, Sullivan M, Franke G, Peczkowska M, Harsch I, Schott M, Gabbert HE, Valimaki M, Preuss SF (2010). Pathogenicity of DNA variants and double mutations in multiple endocrine neoplasia type 2 and von Hippel–Lindau syndrome. Journal of Clinical Endocrinology and Metabolism.

[25] Upadhyaya M, Han S, Consoli C, Majounie E, Horan M, Thomas NS, Potts C, Griffiths S, Ruggieri M, von Deimling A (2004). Characterization of the somatic mutational spectrum of the neurofibromatosis type 1 (NF1) gene in neurofibromatosis patients with benign and malignant tumors. Human Mutation.

[26] Spurlock G, Griffiths S, Uff J, Upadhyaya M (2007). Somatic alterations of the NF1 gene in an NF1 individual with multiple benign tumours (internal and external) and malignant tumour types. Familial Cancer.

[27] McLendon R, Friedman A, Bigner D, Van Meir EG, Brat DJ, Mastrogianakis GM, Olson JJ, Mikkelsen T, Lehman N, Cancer Genome Atlas Research Network (2008). Comprehensive genomic characterization defines human glioblastoma genes and core pathways. Nature.

[28] Buffet A, Venisse A, Nau V, Roncellin I, Boccio V, Le Pottier N, Boussion M, Travers C, Simian C, Burnichon N (2012). A decade (2001–2010) of genetic testing for pheochromocytoma and paraganglioma. Hormone and Metabolic Research.

[29] Eisenhofer G, Vocke CD, Elkahloun A, Huynh TT, Prodanov T, Lenders JW, Timmers HJ, Benhammou JN, Linehan WM, Pacak K (2012). Genetic screening for von Hippel–Lindau gene mutations in non-syndromic pheochromocytoma: low prevalence and false-positives or misdiagnosis indicate a need for caution. Hormone and Metabolic Research.

[30] Ceolin L, Siqueira DR, Ferreira CV, Romitti M, Maia SC, Leiria L, Crispim D, Prolla PA, Maia AL (2012). Additive effect of RET polymorphisms on sporadic medullary thyroid carcinoma susceptibility and tumor aggressiveness. European Journal of Endocrinology.

[31] Jafri M, Maher E (2011). GENETICS IN ENDOCRINOLOGY: The genetics of phaeochromocytoma – using clinical features to guidegenetic testing. European Journal of Endocrinology.

[32] Ross JS, Cronin M (2011). Whole cancer genome sequencing by next-generation methods. American Journal of Clinical Pathology.

[33] Roychowdhury S, Iyer MK, Robinson DR, Lonigro RJ, Wu YM, Cao X, Kalyana-Sundaram S, Sam L, Balbin OA, Quist MJ (2011). Personalized oncology through integrative high-throughput sequencing: a pilot study. Science Translational Medicine.

[34] Tran B, Dancey JE, Kamel-Reid S, McPherson JD, Bedard PL, Brown AM, Zhang T, Shaw P, Onetto N, Stein L (2012). Cancer genomics: technology, discovery, and translation. Journal of Clinical Oncology.

[35] Mirnezami R, Nicholson J, Darzi A (2012). Preparing for precision medicine. New England Journal of Medicine.

[36] Klee EW, Hoppman-Chaney NL, Ferber MJ (2011). Expanding DNA diagnostic panel testing: is more better?. Expert Review of Molecular Diagnostics.

[37] Rothberg JM, Hinz W, Rearick TM, Schultz J, Mileski W, Davey M, Leamon JH, Johnson K, Milgrew MJ, Edwards M (2011). An integrated semiconductor device enabling non-optical genome sequencing. Nature.

[38] Fromer M, Moran JL, Chambert K, Banks E, Bergen SE, Ruderfer DM, Handsaker RE, McCarroll SA, O'Donovan MC, Owen MJ (2012). Discovery and statistical genotyping of copy-number variation from whole-exome sequencing depth. American Journal of Human Genetics.

[39] Diaz LA, Williams RT, Wu J, Kinde I, Hecht JR, Berlin J, Allen B, Bozic I, Reiter JG, Nowak MA (2012). The molecular evolution of acquired resistance to targeted EGFR blockade in colorectal cancers. Nature.

